# Silver diamine fluoride for managing carious lesions: an umbrella review

**DOI:** 10.1186/s12903-019-0830-5

**Published:** 2019-07-12

**Authors:** Nassar Seifo, Heather Cassie, John R. Radford, Nicola P. T. Innes

**Affiliations:** School of Dentistry, University of Dundee, Park Place, Dundee, DD1 4HR UK

**Keywords:** Silver diamine fluoride, Systematic review, Overview, Umbrella review

## Abstract

**Background:**

This umbrella review comprehensively appraised evidence for silver diamine fluoride (SDF) to arrest and prevent root and coronal caries by summarizing systematic reviews. Adverse events were explored.

**Methods:**

Following Preferred Reporting Items for Systematic Reviews and Meta-analysis (PRISMA) guidelines, PubMed, Embase, Cochrane Library, PROSPERO register and Joanna Briggs Institute Database of Systematic Reviews were searched for systematic reviews investigating SDF for caries prevention or arrest (1970–2018) without language restrictions. Systematic reviews were selected, data extracted, and risk of bias assessed using ROBIS by two independent reviewers, in duplicate. Corrected covered area was calculated to quantify studies’ overlap across reviews.

**Results:**

Eleven systematic reviews were included; four focussing on SDF for root caries in adults and seven on coronal caries in children. These cited 30 studies (4 root caries; 26 coronal caries) appearing 63 times. Five systematic reviews were of “low”, one “unclear” and five “high” risk of bias. Overlap of studies was very high (50% root caries; 17% coronal caries). High overlap and heterogeneity, mainly comparators and outcome measures, precluded meta-analysis. Results were grouped by aim and outcomes to present an overview of direction and magnitude of effect. SDF had a positive effect on prevention and arrest of coronal and root caries, consistently outperforming comparators (fluoride varnish, Atraumatic Restorative Treatment, placebo). For root caries prevention, the prevented fraction (PF) was 25–71% higher for SDF compared to placebo (two systematic reviews with three studies) and PF = 100–725% for root caries arrest (one systematic review with two studies). For coronal caries prevention, PF = 70–78% (two systematic reviews with two studies) and PF = 55–96% for coronal caries arrest (one systematic review with two studies) with arrest rates of 65–91% (four systematic reviews with six studies). Eight systematic reviews reported adverse events, seven of which reported arrested lesions black staining.

**Conclusion:**

Systematic reviews consistently supported SDF’s effectiveness for arresting coronal caries in the primary dentition and arresting and preventing root caries in older adults for all comparators. There is insufficient evidence to draw conclusions on SDF for prevention in primary teeth and prevention and arrest in permanent teeth in children. No serious adverse events were reported.

**Electronic supplementary material:**

The online version of this article (10.1186/s12903-019-0830-5) contains supplementary material, which is available to authorized users.

## Background

Dental caries continues to be one of the most prevalent chronic diseases in the world, affecting people across all age groups and countries [1]. Carious lesions can be both prevented and arrested using fluoride-based materials such as professional applied varnishes [2, 3]. Silver diamine fluoride (SDF) was cleared by the Food and Drug Administration in the United States in 2014 [4] with growing interest in its use supported by reports of its effectiveness [5–7]. Silver and fluoride in an alkaline solution act synergistically to arrest carious lesions through a variety of mechanisms [8].

By assessing studies, systematic reviews have explored SDF’s effectiveness to prevent and arrest carious lesions. The ideal systematic review on which to base a clinical decision or guideline would be externally and internally valid, use high-quality methodology, comprehensively include all evidence and carry out a meta-analysis [9]. However, there is no single systematic review of obvious higher quality and recency that should be prioritised in decision-making by those considering adding SDF to their clinical treatment options for patients.

Umbrella reviews also known as systematic reviews of systematic reviews, or overview of systematic reviews, are a relatively new methodology [10, 11]. They filter information by systematically synthesising material from related systematic reviews of an intervention for multiple outcomes. This type of data synthesis allows information required by decision and policy makers to be more accessible and any research gaps to be are identified [12–14]. We have taken an approach, using the Joanna Briggs Institute methodology [15], Cochrane guidance (Becker and Oxman [17]) and recommendations from a recent Cochrane symposium [16], to conduct a transparent review of systematic reviews of SDF.

This umbrella review aimed to provide a low-bias, comprehensive assessment on what the evidence from systematic reviews tells us about using SDF for management of carious lesions in children and adults.

## Objectives

To assess systematic reviews, with or without meta-analyses, of SDF’s effectiveness for:

1. The breadth of evidence assessed in the systematic reviews (systematic reviews’ characteristics and characteristics of their included studies);
2. The risk of bias of the systematic reviews;
3. The arrest and prevention of root and coronal carious lesions in primary and permanent teeth;
4. Adverse events and side effects associated with SDF application.

## Methodology

The protocol was registered in PROSPERO (CRD42017070063) and followed Joanna Briggs Institute [15] and Cochrane methodology [16, 17].

### Inclusion criteria

Systematic reviews with/without meta-analysis investigating SDF (any concentration and frequency) compared with active comparators, placebo and no treatment, for arresting or preventing coronal or root carious lesions in children and adults with or without carious lesions in primary and/or permanent teeth.

### Exclusion criteria

Primary studies investigating SDF or reviews that did not meet the definition of systematic reviews i.e. included a thorough plan and search strategy developed in advance; and aimed to minimise bias by including, appraising, and synthesizing all relevant studies [18].

### Databases and search strategy

We searched databased that contained systematic reviews of health interventions: PubMed, Embase, Cochrane Database of Systematic Reviews and Joanna Briggs Institute Database of Systematic Reviews and Implementation Reports. We also searched the PROSPERO database to allow identification of any forthcoming systematic reviews. These were all searched between 1970 (when SDF was first investigated) and June 2018.

Searches were built around the key words: “silver diamine fluoride” OR “silver diammine fluoride” OR “diamine silver fluoride” OR “diammine silver fluoride” OR “silver fluoride” AND “caries” OR “carious” OR “decayed” OR “cavity” (Additional file 1). AND “review” OR “meta-analysis” was included for databases without a predefined search filter for review articles. No additional search for primary studies was conducted. No language restrictions were applied. Bibliographies of retrieved papers were manually screened to identify additional potential reviews for inclusion.

### Reviews selection process

The search results were imported to Endnote, and duplicates removed. Titles/abstracts screening was performed independently and in duplicate by two reviewers. Full texts of publications considered potentially eligible were retrieved and assessed independently and in duplicate. Where there were discrepancies, a third reviewer was consulted, with discussion until agreement.

### Data collection and synthesis

A standardised data extraction tool was developed a priori and refined based on pilot testing. The data extracted included specific details, such us search strategy, PICO items, objectives, number of included studies (Additional files 2A/B and 3A/B). Two independent reviewers extracted data in duplicate. Root caries systematic reviews and coronal caries systematic reviews were analysed separately because their target populations were different with root caries studies focused on older adults and coronal caries studies on children, and they included different studies with no overlapping included studies. For systematic reviews investigating other interventions alongside SDF, only SDF data were considered.

The breadth of evidence and adverse events assessed in the systematic reviews were summarised narratively through data tables of the review characteristics. To analyse the effectiveness of SDF for managing carious lesions, synthesis of similar outcome measures would have had to be carried out to compare these across comparator interventions and where possible meta-analyses would be carried out.

#### Analysis of the degree of overlap in studies

**Fig. 1:**
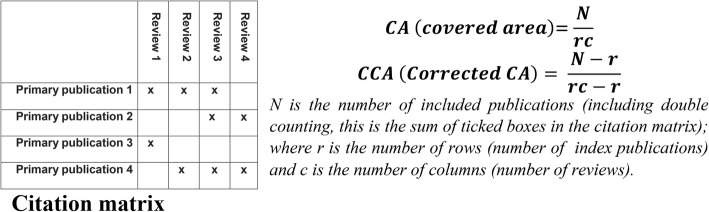
Citation matrix and calculation formulae. CA, covered area; CCA, corrected covered area. 0–5 = slight; 6–10 = moderate; 11–15 = high; over 15 = very high. (Reproduced with the author’s permission) [19]

To determine the overlap in studies across the systematic reviews, citation matrices were generated and “Corrected Covered Areas” (CCAs) were calculated (Fig. 1). CCA = 0–5; slight, 6–10; moderate, 11–15; high, and > 15; very high overlap [19].

#### Reviews’ risk of bias

Two reviewers assessed risk of bias within systematic reviews independently and in duplicate using Risk of Bias in Systematic reviews (ROBIS) [20]. This assesses the systematic reviews across three areas; 1) relevance of the review, 2) identifying concerns within the systematic review process under four domains; study eligibility criteria, identification and selection of studies, data collection and study appraisal, and synthesis and findings and 3) judging risk of bias. These are then considered together to give a “low”, “high” or “unclear” risk of bias score. Scoring discrepancies were resolved through discussion until consensus was reached. Authors were contacted where clarification was required.

## Results

**Fig. 2:**
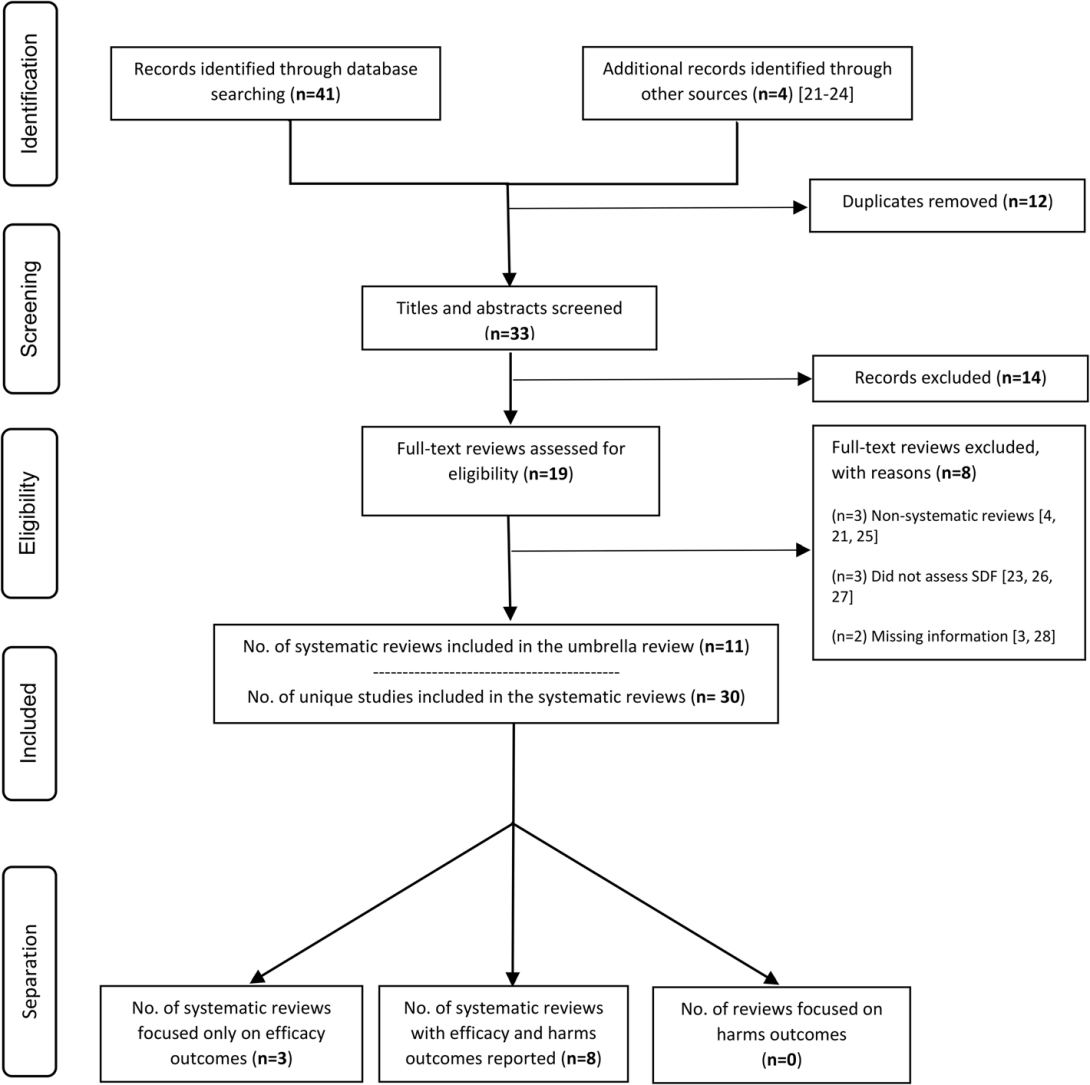
Flow diagram of identification and reviews selection

Figure 2 shows the flow of reviews through searching and assessing. The initial searches yielded 41 potential reviews. Twelve duplicates were removed, and four additional publications added from screening bibliographies resulting in 33 potentially eligible reviews. Following title and abstract screening, 14 papers were excluded and a further eight [3, 4, 21, 23, 25–28] after assessing full texts. Therefore, 11 systematic reviews were included, reporting on 63 studies in total, 30 out of which, were unique.

### Breadth and comprehensiveness of the evidence

Four systematic reviews focussed on root caries [24, 29–31] and seven on coronal caries [5, 22, 32–36]. They included 30 studies (4 root caries; 26 coronal caries). Characteristics of the systematic reviews and studies are summarised below. See Additional files 2A/B, 3A/B and 4 for further details.

#### Characteristics of the reviews

Most systematic reviews covered a defined timeframe, ranging from 1946 to 2017. However, one narrowed it to 2005 to 2015 [33]. PubMed, Cochrane Library and Embase databases were searched most frequently. One systematic review explored grey literature, dissertations and theses [32]. Two systematic reviews searched for on-going trials, dissertations and theses [31, 35]. Only three systematic reviews did not impose any language restrictions [31, 32, 35]. Five systematic reviews restricted language to English [5, 29, 30, 33, 34]. One included English, Spanish and Portuguese [36], one included English and German [24] and one included Japanese, Chinese, English, Portuguese and Spanish [22].

Six systematic reviews considered only children [5, 22, 32–35], whereas, one did not specify an age group indicating “humans” [36]. These seven focused on coronal caries. Two systematic reviews included older adults [29, 30] while two specified adults with exposed root surface [24, 31]. These four systematic reviews focussed on root caries.

Seven systematic reviews included studies investigating SDF alone rather than other interventions [22, 30–33, 35, 36] and four investigated additional agents [5, 24, 29, 34]. Six systematic reviews did not specify a comparison to the intervention [5, 22, 29, 30, 33, 34]. Three systematic reviews included studies comparing the intervention to no treatment, placebo or other interventions [24, 31, 32, 35]. One systematic review compared SDF to fluoride varnish [36].

Seven systematic reviews investigated the effect of SDF on coronal carious lesions with four focussing on lesions arrest [5, 22, 32, 34], one on prevention only [35] and two on both prevention and arrest [33, 36]. Of the four systematic reviews that investigated root caries, one focused on carious lesions prevention only [29], three explored carious lesions prevention and arrest [24, 30, 31]. Eight systematic reviews reported adverse events and side effects associated with SDF treatment [5, 22, 30, 31, 33–36].

The systematic reviews used six different outcome measures: % success rates [5, 22, 29, 34]; prevented fraction (PF) [30, 31, 35, 36]; number needed to treat (NNT) [30, 36]; weighted mean difference (WMD) [31, 35]; mean difference (MD) [24] and risk ratio (RR) [32]. The outcome measurement was not clear in one systematic review that did not synthesise results from included studies but presented the original reported data [33]. Six conducted meta-analyses to synthesize the findings [22, 24, 31, 32, 34, 35].

Eight systematic reviews used Cochrane risk of bias assessment tool or a simplified version based on its recommendations [5, 22, 24, 31–35]. One used Jadad,1998 [36], and one used the critical appraisal sheet for randomized controlled trials (RCTs) from Oxford Centre for Evidence-Based Medicine [30]. However, one systematic review did not evaluate the included studies [29]. Regarding grading the quality of evidence, two systematic reviews used GRADE [24, 32].

#### Characteristics of the studies included in the reviews

The number of studies contained within the systematic reviews varied widely; three systematic reviews included one or two RCTs [24, 29, 36], while others included seven or more. Gao included seven RCTs focussing on SDF in one systematic review [34] and 19 prospective clinical trials in another [22].

Five systematic reviews did not state the studies’ country of origin [24, 29, 32, 34, 36]. Eight studies were conducted in Brazil, seven in Hong Kong, six in China and four in Japan. One study was conducted in each of the following countries: Nepal, Philippines, Cuba, Argentina and Turkey.

The first trial investigating SDF was published in 1969 [37]. Despite all systematic reviews apart from one systematic review [33] searching earlier timeframes, only one retrieved studies published before 2002 [22].

The root caries studies were of high quality and at low risk of bias, while for coronal caries studies; the reliability of those conducted before 2002 was relatively low, while studies after that were of better quality.

### Systematic reviews’ risk of bias

Five systematic reviews were at high [5, 24, 29, 30, 36], five were at low [22, 31, 32, 34, 35] and one was at unclear [33] risk of bias (Additional file 3A/B).

As SDF is popular in non-English speaking countries and studies were often reported in non-English journals, limiting to English language reduced the comprehensiveness of included studies and immediately placed significant bias within those systematic reviews [5, 29, 30, 33, 34]. In addition, the absence of a priori designed protocol, affected the risk of bias score for eight systematic reviews since there was no indication that predefined analyses were followed [5, 22, 24, 29, 30, 33, 34, 36].

Three systematic reviews did not report whether study selection had been undertaken independently and in duplicate [30, 33]. Moreover, two did not report whether bibliography screening or other manual search methods were used [5, 33].

It was unclear in three systematic reviews whether data collection had been undertaken, independently and in duplicate [30, 33, 34]. All systematic reviews, except one which did not appraise the included studies [29], appraised the studies using appropriate criteria.

### Findings of the reviews

In umbrella reviews, the role of the reviewer is to appraise the evidence from the systematic reviews and not the studies. The appropriateness of re-analysis of studies’ data has been debated but it is generally agreed that where novel analyses are the aim, a review of studies should be undertaken rather than an overview of reviews [13, 38]. systematic reviews outcome and outcome measures heterogeneity meant that meta-analysis was not appropriate. However, we combined systematic review results together to present an overview of direction and magnitude of effect where there was the same outcome measure [39]. (Additional files 3A/B and 4).

#### Root carious lesions management in adults

For root carious lesions prevention and arrest, all four systematic reviews compared SDF to placebo and found the direction of effect favoured SDF i.e. there were more prevented and arrested lesions with the use of SDF.

##### Carious lesions prevention

For root carious lesions prevention, the success rates were 72% higher for 38% SDF compared to placebo based on one high risk of bias systematic review including one study [29]. The MD for changes in DMFRS/DFRS was − 0.33 (95% CI = − 0.39, − 0.28) for 38% SDF compared to placebo based on one high risk of bias systematic review with meta-analysis of two studies [24]. The PF was 25–71% for 38% SDF compared to placebo based on one low [31] and one high [30] risk of bias systematic reviews including four studies.

##### Carious lesions arrest

For root carious lesions arrest the PF was 100 to 725% higher for 38% SDF than placebo based on a single high risk of bias systematic review [30] with data from two studies. One low risk of bias systematic review reported that 38% SDF was significantly more effective than placebo in arresting root carious lesions (pooled results were not calculated) [31]. One systematic review found that SDF can be efficacious to decrease progression of root carious lesions (no numeric results reported) [24].

#### Coronal carious lesions management in children

For coronal carious lesions prevention and arrest, all seven systematic reviews focused mainly on primary dentition and all reported that SDF outperformed the comparators regardless of the outcome measure.

##### Carious lesions prevention

Coronal carious lesions prevention was reported in three systematic reviews; one at low risk of bias focused only on the primary dentition [35] and one at unclear [33] and one at high [36] risk of bias focused on the primary dentition and first permanent molars. The PF for 38% SDF compared to placebo ranged from 70 to 78% in the primary dentition based on two systematic reviews [35, 36] including two studies and was 64% in the permanent first molars based on one systematic review [36] with one study [6].

For fluoride varnish compared to placebo in the primary dentition the PF was 54% based on one systematic review [35] with one study [40]. The same systematic review reported that glass ionomer cement was more effective than 30% SDF at 12 months, PF = − 6%, but the difference was not statistically significant.

One systematic review presented studies’ original results and concluded that SDF showed potential as a caries preventive treatment in the primary dentition and for first permanent molars [33].

##### Carious lesions arrest

Coronal carious lesions arrest was reported in six systematic reviews; three at low [22, 32, 34], two at unclear [33] and one at high [5, 36] risk of bias systematic reviews including eight studies.

The reported 38% SDF arrest rates in the primary dentition ranged from 65 to 91% based on three systematic reviews [5, 22, 34]. These were 38 to 44% for fluoride varnish, 39 to 82% for glass ionomer cement, and 34% for placebo. The PF based on one systematic review [36] with two studies ranged from 55 to 96% in favour of 38% SDF when compared to fluoride varnish or placebo in primary dentition. However, this was 100% for permanent first molars based on one study [6]. The RRs were 1.66 for SDF compared to fluoride varnish or Atraumatic Restorative Treatment and 2.54 compared with placebo/no treatment based on one systematic review which focused only on the primary dentition [32] and including two studies. One systematic review presented the studies’ original results and concluded that SDF at concentrations of 30 and 38% is more effective than other strategies in arresting coronal carious lesions in primary dentition [33].

##### Adverse events and side effects

Eight systematic reviews reported adverse events and side effects associated with SDF [5, 22, 30, 31, 33–36]. The main side effect reported was black staining of the carious lesions although older adults rarely complained about that. Similarly, the discoloration was acceptable in children, concerning 7% of participants in one study [40].

Adverse events were classified into two categories according to the FDA definition and classification of adverse events [41]; suspected adverse reaction or adverse event where there is a reasonable possibility that this is caused by the drug, and serious adverse event or serious suspected adverse reaction. An adverse event is considered “serious” if it causes death, a life-threatening event or in-patient hospitalisation.

Regarding suspected adverse reactions, reversible, small, mildly painful white lesions in oral mucosa, due to inadvertent contact with SDF, were reported; these healed uneventfully within 48 h. There was no difference in pulpal irritation incidence between the control and experimental groups. A metallic taste or burning sensation was not reported in any of the studies. No serious adverse events, such as allergic reactions or toxicity were reported.

## Discussion

We identified 11 systematic reviews (of 30 studies) investigating SDF for carious lesions prevention and/or arrest; seven focused on coronal caries in children, and four on root caries in adults. This is a high ratio of studies to systematic reviews with several published in the last few years indicating that no single systematic review seems to have incorporated all the evidence and comprehensively covered the topic. We have attempted to address this by systematically appraised the evidence from the systematic reviews using a transparent methodology and have found that all systematic reviews, despite variability in methodology, found SDF to be more effective for carious lesions prevention and arrest than any of the comparators.

When interpreting the results of this umbrella review, it should be kept in mind that the individual studies included in the systematic reviews are not scrutinised. Therefore, our conclusions rely on the interpretation of the systematic reviews’ authors. This is in line with the accepted umbrella review methodology and capitalises on the fact the original studies had their qualities appraised within the systematic review in which they were reported.

**Fig. 3:**
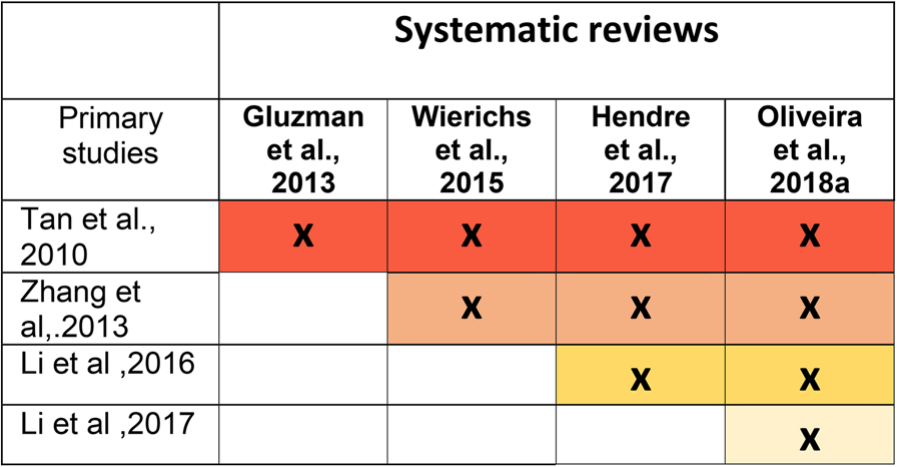
Citation matrix for reviews assessed the effectiveness of SDF for root carious lesions management

**Fig. 4:**
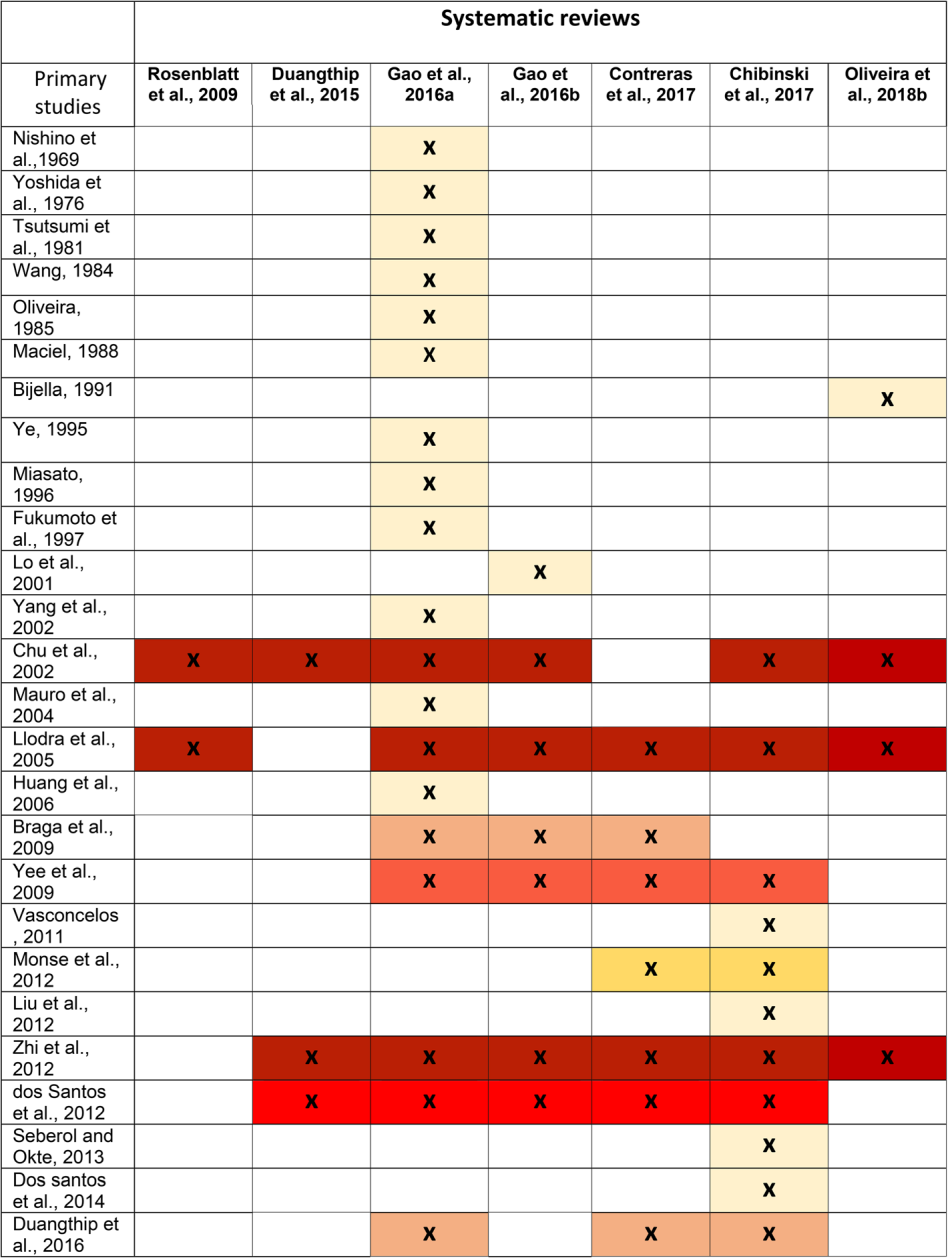
Citation matrix for reviews assessed the effectiveness of SDF for coronal carious lesions management

With 11 systematic reviews including 30 studies and within this, four systematic reviews focussing on root caries including four studies, the overlap in studies across systematic reviews was very high in both matrices [19]. The CCA was 0.5 for root caries systematic reviews (50% overlap) and 0.17 for coronal caries systematic reviews (17% overlap) (Figs. 3 and 4). This means that a large number of studies appeared several times across the systematic reviews. Consequently, repeated studies would have had unintentionally stronger weighting in any meta-analyses. This, together with heterogeneity of comparators and outcome measures limited synthesis of the results and precluded meta-analyses. In addition, in line with standard Umbrella review methodology, each meta-analysis was not re-calculated to confirm validity. However, allowing for these caveats, this umbrella review is the first such review systematically summarising the current evidence for the effectiveness of SDF for carious lesions prevention and arrest. It followed a systematic approach that included a comprehensive search strategy of five databases with independent, duplicate systematic review selection and data extraction and an accepted method to assess risk of bias.

Many SDF studies have been set in non-English speaking countries such as China and Brazil. Thirteen out of the 30 unique studies were published in non-English languages. However, five systematic reviews excluded non-English studies, and this is likely to have introduced bias into their dataset, analyses and conclusions.

There was wide variability in the number of included studies ranging from only two [36] up to 19 [22] even when they investigated similar interventions/comparators, aims and outcomes as well as including similar study designs. Without further investigation, it was not possible to determine the reason for this, however there is an interesting difference between the coronal and root caries systematic reviews. In the root caries systematic reviews, Fig. 3 shows that when the systematic review was more recent, there were more studies included, and all seem to have been captured by the searches. This contrasts with Fig. 4 which shows an irregular pattern of study inclusion for the coronal caries systematic reviews. This pattern is not explained by the search timeframe or year of publication as more recent systematic reviews would be expected to include more recently published studies in addition to capturing all previous ones. Although it was not within the remit of this review to undertake a full exploration of the reasons for inclusion and exclusion of studies in the systematic reviews, it does not seem to be explained by differing inclusion/ exclusion criteria or other methodological decisions. Some of these findings might offer insight into this and inform future work looking at the quality of systematic reviews. For example, one study [42] investigated the effectiveness of Nano Silver Fluoride (NSF) for preventing and arresting carious lesions in children. It was included in a review investigating SDF [32] and the justification given, on contacting the authors, was because NSF contained the same components as SDF; this effect remained the same even when silver fluoride was chemically treated to obtain nanoparticles of silver. However, other investigators excluded this study, possibly because they viewed NSF as different from SDF. Alternatively, they did not detect this paper in their search. On the other hand, the same systematic review [32] excluded a study [43] investigating SDF in arresting occlusal carious lesions in first permanent molars because the method of evaluation was based on qualitative scores. However, it was included in three other systematic reviews [22, 33, 34]. Although this finding is incidental and was not one of the aims of the umbrella review, it is notable and perhaps worth investigating further. It is not possible to quantify this in terms of quality of the individual reviews and this is perhaps a limitation of umbrella reviews. The usefulness of the risk of bias scoring tools is also questioned with these findings. If one of these systematic reviews was assessed individually, it could score at low risk of bias and be considered as a good systematic review for basing policy on, yet there could be many studies not included and a resulting hidden high risk of bias with no insight into the consequences of omitting certain studies. Poor decisions to include or exclude studies could easily go undetected. The lack of comprehensiveness in the systematic reviews is not fully related to low quality and only revealed by comparing the systematic reviews.

These findings highlight the need for meticulous attention to be paid during studies’ selection processes and for those appraising reviews to be aware that this might be a shortcoming not detected during quality appraisal. It also stresses the need for systematic reviews to provide data justifying the exclusion of each study, and not simply report the total number of excluded studies with overall reasons. This would help clarify whether all possible studies were found through searching and rule out selection bias. So, even systematic reviews at low risk of bias, according to ROBIS tool, might fail to provide healthcare decision makers with accurate evidence depending on how they include or exclude studies relevant to their question. For SDF, all the systematic reviews pointed to evidence of a positive effect rather than conflicting results depending on which systematic review was looked at.

For the root caries systematic reviews, the main limitation was around conclusions being based on a limited number of included studies (one systematic review drew conclusion based on only one study). This demonstrates the need for more well-conducted RCTs investigating SDF for root carious lesions management. Imposing language restrictions and the absence of a priori designed protocol had affected the risk of bias in three out of the four systematic reviews. One systematic review brought dentine hypersensitivity into their conclusions even though this was neither included in the search nor discussed through the systematic review [30].

For the coronal caries systematic reviews, a larger number of studies was included in the systematic reviews. However, the quality of included studies varied with those conducted before 2002 being of low reliability. Moreover, the methodology and outcome measurements varied between studies which made combining the results challenging. This supports the need for designing a standardised methodology and following a core outcome set, if possible, for studies in reporting their results, in order to enable systematic reviews in synthesising the evidence from all available relevant studies.

Further details about the limitations of each included systematic review can be found in Additional file 3A/B.

Another finding worth noting was that the search in the PROSPERO register retrieved six ongoing, apparently unfinished systematic reviews. Three were completed and published however their statuses had not been updated in PROSPERO [31, 32, 35]. It was possible however to retrieve these from searches in PubMed and Embase.

Overall, all systematic reviews reported that SDF was effective in managing carious lesions. However, earlier ones tended to overstate conclusions around SDF’s effectiveness given the limited number of trials they were based on, and the systematic reviews’ high risk of bias. More recent systematic reviews reported increasing numbers of trials and were of lower risk of bias.

For root carious lesions *prevention and arrest*, the systematic reviews were based on only four clinical trials. However, all trials were assessed as high quality in the systematic reviews.

There was a large variability in the number of studies included in the coronal caries systematic reviews and the reasons for this were unclear.

For coronal carious lesions *prevention*, it is noteworthy that the number and quality of studies included in the systematic reviews was low which questions the evidence base around SDF for coronal carious lesions prevention.

For coronal carious lesions *arrest*, an increased number of systematic reviews have reported stronger evidence to support SDF use in the primary dentition. There is insufficient evidence to draw conclusions for its use in permanent teeth in children as there are so few studies.

## Conclusions

Although there are not a large number of clinical trials, there is a consistent and progressively strengthening body of research that supports SDF’s effectiveness for arresting coronal carious lesions in children in the primary dentition and arresting and preventing root carious lesions in older adults. However, the evidence base around SDF for preventing coronal carious lesions in children was questionable based on the number and quality of studies. Moreover, there are too few studies and insufficient evidence to draw conclusions on the use of SDF in permanent teeth in children.

## Additional files

Additional file 1: PubMed search strategy. (DOCX 22 kb)

Additional file 2: Root and coronal caries reviews characteristics. (DOCX 47 kb)

Additional file 3: Root and coronal caries reviews' findings and quality appraisal. (DOCX 168 kb)

Additional file 4: Characteristics of reviews related to outcomes, outcome measures and results. (DOCX 40 kb)

## Data Availability

The authors declare that the data supporting the findings of this study are available within the article and its supplementary information files.

## Abbreviations

CCA: Corrected covered area
FDA: Food and Drug Administration
MD: Mean difference
NNT: Number needed to treat
NSF: Nano silver fluoride
PF: Prevented fraction
RR: Risk ratio
SDF: Silver diamine fluoride
WMD: Weighted mean difference
