# Supplementary material for: Silver diamine fluoride for managing carious lesions: an umbrella review
Source: BMC Oral Health. 2019 Jul 12;19:145. doi: 10.1186/s12903-019-0830-5 (PMC6626340; doi:10.1186/s12903-019-0830-5)
Supplement: Supplementary file 1 — PubMed search strategy. (DOCX 22 kb) [file 12903_2019_830_MOESM1_ESM.docx]

Nassar Seifo, Heather Cassie, John R Radford, Nicola P T Innes

| **Additional file 1. PubMed search strategy** |
| --- |
| 1. (silver diam?ine fluoride[Title/Abstract]) 2. (diam?ine silver fluoride[Title/Abstract]) 3. (silver fluoride[Title/Abstract]) 4. (cari*[Title/Abstract]) 5. (decay*[Title/Abstract]) 6. (cavit*[Title/Abstract]) 7. (Meta-Analysis[ptyp]) 8. (systematic[sb]) 9. #1 OR #2 OR #3 10. #4 OR #5 OR #6 11. #7 OR #8 12. #9 AND #10 AND #11 |
