# Supplementary material for: Silver diamine fluoride for managing carious lesions: an umbrella review
Source: BMC Oral Health. 2019 Jul 12;19:145. doi: 10.1186/s12903-019-0830-5 (PMC6626340; doi:10.1186/s12903-019-0830-5)
Supplement: Supplementary file 2 — Root and coronal caries reviews characteristics. (DOCX 47 kb) [file 12903_2019_830_MOESM2_ESM.docx]

| **Additional file 2A. Root caries reviews’ characteristics** | | | | | | | | | |
| --- | --- | --- | --- | --- | --- | --- | --- | --- | --- |
| **Authors/ Title/ Citation/ Funding source** | **Year** | **Aim or research question** | **Search strategy** | **Participants** | **Interventions** | **Comparators** | **Outcomes** | **Meta-analysis** | **Instruments used to assess studies/evidence** |
| R. Gluzman, R.V Katz, B.J. Frey, R. McGowan  **Prevention of root caries: a literature review of primary and secondary preventive agents**  Spec Care Dentist 33(3): 133-140  *The American Dental Association and The National Institute for Dental Craniofacial Research at NIH* | 2013 | “To summarize the effectiveness of seven leading root caries preventive agents and provide recommendations for use of those agents in clinical practice with older adults and vulnerable elderly.” | ***Databases:***  PubMed/Medline and Cochrane Library  ***Other approaches:***  Reviewing reference lists and search for the most recent publications in main dental journals to compensate for the delay in transfer to electronic database  ***Search period:***  Jan 1979 – Jul 2010  ***Language restriction:***  *Only English papers were included* | Older adults | Fluoride, chlorhexidine, xylitol, amorphous calcium phosphate, sealants, saliva stimulators, and SDF. | Not stated | **Outcomes:**  Reduction in root caries incidence or root caries arrest  **Outcomes measurement:**  The percentage reduction in root caries was calculated. | No | There was no assessment of the quality of included studies |
| R.J. Wierichs, H. Meyer-Lueckel  **Systematic review on noninvasive treatment of root caries lesions**  Journal of Dental Research, Vol. 94(2) 261-271  *The authors and their institution (RWTH Aachen University)* | 2015 | “To systematically retrieve and analyze clinical studies investigating chemical agents to reduce the initiation of root caries lesion and/or increase their inactivation.” | ***Databases:***  PubMed, EMBASE, and Cochrane Library  ***Other approaches:***  Cross-referencing  ***Search period:***  Jan 1947- May 2014  ***Language restriction:***  English and German papers were included | “Humans who retained a minimum of 1 natural tooth with exposed root surfaces with or without root caries lesions” | Preventive dental regimes (e.g., oral health instruction) and/or 1 or more chemical agents applied on 1 or more occasion by a dental professional or self-applied by the patient. | placebo, positive interventions; e.g., Duraphat varnish), or standard therapy” | **Outcomes:**  Clinical or radiographic visible changes of active or inactive root caries.  **Outcomes measurement:**  Mean differences were calculated for changes in DMFRS/DFRS | Yes | “Risk of bias assessment was performed according to guidelines outlined by the Cochrane Collaboration.”  “Grading of evidence was performed according to the GRADE network levels, based on Grade Profiler 3.6.”  “Publication bias was assessed by funnel plots.” |
| A.D Hendre, G.W. Taylor, E.M. Chavez, S. Hyde  **A systematic review of silver diamine fluoride: Effectiveness and application in older adults**  Gerodontology; 34:411-419  *An unrestricted honorarium from the American Dental Association’s National Elder Care Advisory Committee of the Council on Access, Prevention and Interprofessional Relations* | 2017 | “To examines the effectiveness of silver diamine fluoride (SDF) in the management of caries in older adults.” | ***Databases:***  PubMed, PubMed Clinical Queries, EMBASE, the American Dental Association’s Evidence-Based Dentistry Website, Cochrane Library, Web of Science, repository of the Journal of the American Dental Association and Google Scholar.  ***Other approaches:***  Hand search of bibliographies  ***Search period***  1946 to November 2015 with monthly reruns of search terms in PubMed through August 2016:  ***Language restriction:***  Only English papers were included | Adults | SDF | Not stated | **Outcomes:**  Caries prevention, arrest or remineralization.  **Outcomes measurement:**  The effectiveness of SDF was measure using the following parameters: NNT, PF, relative risk, arrest rate and mean number of new carious surfaces and mean number of arrested root surfaces. | No | The critical appraisal worksheet for randomized controlled trials from the Oxford Centre for Evidence-based Medicine (CEBM 2005) provided the framework to assess the quality and risk of bias of the selected articles.  The appraisal worksheet was slightly modified: Question 3b was added to the therapy appraisal for clinical trials to gauge interexaminer calibration. |
| B.H Oliveira, J. Cunha-Cruz, A. Rajendra, R. Niederman  **Controlling caries in exposed root surfaces with silver diamine fluoride: A systematic review with meta-analysis**  J Am Dent Assoc.149(8):671-679.  *The National Institute on Minority Health and Health Disparities of the National Institutes of Health under Award No. R01MD011526 and U24MD006964, and partially funded through a Patient-Cen­tered Outcomes Research Institute Award (PCS-1609- 36824). The Teacher Training Program of the University of the State of Rio de Janeiro also sup­ported this work.* | 2018 | “To assess the effect of SDF in preventing and arresting caries in exposed root surfaces of adults.” | ***Databases:***  Cochrane Library, Embase, MEDLINE via PubMed, Scopus, Web of Science, Latin American and Caribbean Health Sciences Literature, Biblioteca Brasileira de Odontologia, SciELO, Clinical Trials.gov, Brazilian Clinical Trials Registry, European Union Clinical Trials Register, International Standard Randomised Controlled Trials Registry and Current Controlled Trial, Australian New Zealand Clinical Trials Registry and Brazilian database of theses and dissertation  ***Other approaches:***  Cross-referencing from narrative reviews  ***Search period***  No date restrictions to start up to July 2017.  ***Language restriction:***  No restrictions | Adults with exposed root surfaces | SDF | No intervention, placebo, or any cariostatic agent or restorative material | **Outcomes:**  Primary outcome: development of new carious lesions and arrest of existing carious lesions in exposed root surfaces of permanent teeth within at least 12 months after produce application (e.g. 12, 24, 36 months or more of follow-up).  Secondary outcome: any self-reported, caregiver-reported, or professionally diagnosed adverse events.  **Outcomes measurement:**  For caries prevention, the difference in mean caries increment between SDF and control groups, PFs were calculated.  For caries arrest, the difference in mean numbers of arrested lesions between SDF and control groups. | Yes | Cochrane risk of bias assessment tool |

| **Additional file 2B. Coronal caries reviews’ characteristics** | | | | | | | | | |
| --- | --- | --- | --- | --- | --- | --- | --- | --- | --- |
| **Authors/ Title/ Citation/Funding source** | **Year** | **Aim or research question** | **Search strategy** | **Participants** | **Interventions** | **Comparators** | **Outcomes** | **Meta-analysis** | **Instrument used to assess studies/evidence** |
| A. Rosenblatt, T.C.M. Stamford, R. Niederman  **Silver diamine fluoride: a caries “silver-fluoride bullet”**  J Dent Res 88(2):116-125.  *The Fulbright Program and The Forsyth Institute* | 2009 | “Will SDF more effectively prevent caries than fluoride varnish?” | ***Databases:***  MEDLINE, LILACS, EMBASE, the Cochrane Library, and the Brazilian Dental Library  ***Other approaches:***  Reviewing reference lists  ***Search period:***  1966 - Dec 31, 2006  ***Language restriction:***  English, Spanish, or Portuguese papers were included | “Humans” | SDF | Fluoride varnish | **Outcomes:**  Although the research question is about prevention, the study reported both caries prevention and arrest  **Outcomes measurement:**  The prevented fraction (PF) and number needed to treat (NNT) were calculated from the original data. | No | Jadad, 1998 |
| D. Duangthip, M. Jiang, C.H. Chu, E.C.M. Lo  **Non-surgical treatment of dentin caries in preschool children – systematic review**  BMC Oral Health15:44  *Faculty of Dentistry, University of Hong Kong* | 2015 | *“To* systematically review and evaluate the literature on the effectiveness of non-surgical methods in arresting or slowing down the progression of active dentine caries in primary teeth in preschool children.” | ***Databases:***  PubMed, Cochrane Library, and EMBASE  ***Other approaches:***  None  ***Search period:***  1947- Jun 2014    ***Language restriction****:*  Only English papers were included | “Children aged 6 or below who had at least one dentin carious lesion in the primary dentition” | “Various non-surgical interventions such as fluoride agents, dental sealant, resin infiltrant, CHX, xylitol, CPP-APC, ozone and oral health education” | Not stated | **Outcomes:**  Caries arrest, progression or regression. There could be comparisons of outcomes of different nonsurgical approaches, or nonsurgical and surgical approaches  **Outcomes measurement:**  The primary summary measure for reporting in this review was success rates of various treatments. | No | Cochrane risk of bias assessment tool;  Graded for quality using ADA criteria. |
| S.S. Gao, I.S. Zhao, N. Hiraishi, D. Duangthip, M.L. Mei, E.C.M. Lo, C.H. Chu  **Clinical trials of silver diamine fluoride in arresting caries among children: a systematic review**  JDR Clinical & Transitional Research, Vol 1, Issue 3, page(s): 201-210  *The General Research Fund (number: 17107315) of the University Grant Council, Hong Kong* | 2016 | “To investigate the clinical effectiveness of SDF in arresting dental caries among children.” | ***Databases:***  PubMed, EMBASE, Scopus, China National Knowledge Infrastructure (CNKI), Ichushi-web, Biblioteca Virtual en Salud Espana (BVSE) and Biblioteca Virtual em Saude (BVS)  ***Other approaches:***  Manual screening of the bibliographies  ***Search period:***  No limit, the last search was made in Mar 2016  ***Language restriction****:*  English, Chinese, Japanese, Portuguese and Spanish papers were included | Children (primary and permanent teeth) | SDF | With or without control group | **Outcomes:**  Carries arrest  **Outcomes measurement:**  The percentage of dental caries that had become arrested after SDF treatment was calculated if possible. | Yes | “Risk of bias was assessed for each included study from 6 aspects: 1) random sequence generation (selection bias), 2) allocation concealment (selection bias), 3) blinding of outcome assessment (detection bias), 4) incomplete outcome data (attrition bias), 5) selective reporting (reporting bias), and 6) other bias.” |
| S.S. Gao, S. Zhang, M.L. Mei, E.C.M. Lo, C.H. Chu  **Caries remineralisation and arresting effect in children by professionally applied fluoride treatment – a systematic review**  BMC Oral Health 16:12  *The General Research Fund (number: 17107315) of the University Grant Council, Hong Kong* | 2016 | “To investigate the clinical efficacy of professional fluoride therapy in remineralising and arresting caries in children.” | ***Databases:***  PubMed, Cochrane Library, ISI Web of Science and EMBASE  ***Other approaches:***  Manual screening of the bibliographies  ***Search period:***  1948 - 2014  ***Language restriction:***  Only English papers were included | Children (primary and permanent teeth) | Silicon tetrafluoride, fluoride gel, SDF, sodium fluoride or nano-silver fluoride | Not stated | **Outcomes:**  The remineralisation or arresting effect of caries by professional fluoride treatment.  **Outcomes measurement:**  The percentage of remineralised early enamel caries and the percentage of arrested dentine caries were calculated. | Yes | Cochrane risk of bias assessment tool |
| V. Contreras, M.J Toro, A.R. Elias-Boneta, A. Encarnacion-Burgos  **Effectiveness of silver diamine fluoride in caries prevention and arrest: a systematic literature review**  AGD; No. 402, p. 30  *National Institutes of Health Award No. HCTRECD R25MD007607 and HiREC S21MD001830 from the National Institute on Minority Health and Health Disparities* | 2016 | “Is the use of SDF more effective than other strategies in the prevention and/or arrest of carious lesions in children who have caries in primary teeth and/or permanent first molars?” | ***Databases:***  PubMed, Science Direct and Scopus  ***Other approaches:***  *None*  ***Search period:***  Aug 2005 – Jan 2016  ***Language restriction:***  Only English papers were included | Children (primary and permanent teeth) | SDF | Not stated | **Outcomes:**  Mean number of surfaces with new, active, and inactive caries at baseline and follow-up; mean numbers of arrested surfaces; the percentage of caries arrest; and caries increment  **Outcomes measurement:**  Not clear. Only the original data was reported. | No | Risk of bias was assessed using a simplified analysis adapted from the recommendations in the Cochrane Handbook of Systematic Reviews of Interventions |
| A.C. Chibinski, L. M. Wambier, J. Feltrin, A.D Loguercio, D.S Wambier, A. Reis  **Silver Diamine Fluoride Has Efficacy in Controlling Caries Progression in Primary Teeth: A Systematic Review and Meta-Analysis**  Caries Res; 51:527-541  *The National Council for Scientific and Technological Development under grants 304105/2013-9 and 305588/2014-1* | 2017 | “To evaluate the efficacy of silver diamine fluoride (SDF) in controlling caries progression in children when compared with active treatments or placebos.” | ***Databases:***  PubMed, Scopus, Web of Science, the Latin American and Caribbean Health Sciences Literature database (LILACS), the Brazilian Library in Dentistry (BBO) and the Cochrane Library).  The grey literature was explored using the database System for Information on Grey Literature in Europe and Google Scholar. Dissertations and theses were searched using the ProQuest Dissertations and Theses Full Text databases and the Periodicos Capes Theses database.  ***Other approaches:***  Hand search of reference lists  ***Search period:***  No restrictions  ***Language restriction***  No restrictions | Children (primary and permanent teeth) | SDF | placebo or other active treatments | **Outcomes:**  The arrestment of the carious lesion in enamel or dentin.  **Outcomes measurement:**  The outcomes were summarized by calculating the risk ratio and the 95% confidence interval | Yes | The risk of bias tool from the Cochrane Collaboration was used for quality assessment of the studies. The quality of the evidence was evaluated using the GRADE approach. |
| B.H Oliveira, A. Rajendra , A.V Keenan, R. Niederman  **The Effect of Silver Diamine Fluoride in Preventing Caries in the Primary Dentition: A Systematic Review and Meta-Analysis**  Caries Res; 6;53(1):24-32  *The National Institute on Minority Health and Health Disparities of the National Institutes of Health under Award No. R01MD011526 and U24MD006964, and partially funded through a Patient-Cen­tered Outcomes Research Institute Award (PCS-1609- 36824). The Teacher Training Program of the University of the State of Rio de Janeiro also sup­ported this work.* | 2018 | “To investigate whether silver diamine fluoride (SDF) is effective in preventing new caries lesions in primary teeth when compared to placebo or active treatments.” | ***Data Bases***  Cochrane Library, Embase, MEDLINE via PubMed, Scopus, Web of Science, Latin American and Caribbean Health Sciences Literature, Biblioteca Brasileira de Odontologia, SciELO, Clinical Trials.gov, Brazilian Clinical Trials Registry, European Union Clinical Trials Register, International Standard Randomised Controlled Trials Registry and Current Controlled Trial, Australian New Zealand Clinical Trials Registry and Capes Dissertations database  ***Other approaches:***  Cross-referencing from narrative reviews  ***Search period***  No date restrictions to start up to July 2017.  ***Language restriction:***  No restrictions | Children (Primary teeth) | SDF | No intervention, placebo, any topical cariostatic agents, resin or glass ionomer pit and fissure sealants or dental restorative materials; | **Outcomes**  Primary outcome: the development of new dentin caries lesions at surface, tooth, or patient level.  Secondary outcome: any self/parent-reported or professionally diagnosed adverse events.  **Outcomes measurement:**  The difference in mean caries increment between SDF and control groups, PFs were calculated. | Yes | Cochrane risk of bias assessment tool |
