# Supplementary material for: Silver diamine fluoride for managing carious lesions: an umbrella review
Source: BMC Oral Health. 2019 Jul 12;19:145. doi: 10.1186/s12903-019-0830-5 (PMC6626340; doi:10.1186/s12903-019-0830-5)
Supplement: Supplementary file 3 — Root and coronal caries reviews' findings and quality appraisal. (DOCX 168 kb) [file 12903_2019_830_MOESM3_ESM.docx]

| **Additional file 3A. Root caries reviews’ findings and quality appraisal** | | | | | | | | | | | | | | | |
| --- | --- | --- | --- | --- | --- | --- | --- | --- | --- | --- | --- | --- | --- | --- | --- |
| **Systematic review** | **Number of studies**  **Sample size (range)** | **Countries in which studies were conducted** | **Publication date range** | **Review authors’ stated limitations** | **Adverse events** | **Review authors' stated conclusions** | **Quality assessment*** | | | | | | **Overview authors’ additional limitations** | **Overview authors’ comments** |  |
|  |  |  |  |  |  |  | **AR** | **Review Process** | | | | **RoB** |  |  |  |
|  |  |  |  |  |  |  |  | **SEC** | **ISS** | **DCSA** | **SF** |  |  |  |  |
| *Gluzman (2013)*  **Prevention of root caries: a literature review of primary and secondary preventive agents** | 31 studies - one SDF trial.  306 | Not stated**  *Hong Kong* | 2010 | “For the primary prevention studies, the “depth” of evidence is “thin”, since all six of the most effective primary prevention agents or combination of agents were each tested only in a single study.” | Not reported | “For the 1° prevention of root caries the recommended “best choice” is the 38% SDF solution professionally applied annually.” | **√** |  |  |  |  |  | There was no appraisal for the quality of each individual study. Besides, there was no table of the characteristics and summary of the included studies and the language was limited to English only. | Combining the effectiveness for all these different approaches is a very crude way of doing it and the heterogeneity of the studies really limits the conclusion and there should be little confidence in the findings having it done by this way, but the authors have been tasked with coming up with recommendations and forced results from the paper.  The evidence regarding the effectiveness of SDF on root caries is very thin because it is based on only one study. |  |
| *Wierichs and Meyer-Lueckel (2015)*  **Systematic review on noninvasive treatment of root caries lesions** | 30 studies - two SDF trials  572 (266-306) | Not stated**  *Hong Kong 2* | 2010-2013 | Low numbers of clinical trials for each agent (2 SDF studies). | Not reported | SDF vanish may inactivate existing and/or reduce the initiation of root caries lesions. However, results should be interpreted with caution, due to some limitations. | **√** |  |  |  |  |  | Although the authors combined the data, both SDF studies had different application frequencies with Tan quarterly and Zhang annually.  Language was restricted to English and German.  There was no referral to a priori designed protocol.  Although the authors mentioned that 11 studies were not sponsored by manufactures. It is not clear which ones were and which ones were not. | Only 2 English studies were included. Both showed effectiveness for root caries lesions arrest/prevention in favor of SDF.  However, a definite conclusion could not be drawn based on 2 studies. |  |
| *Hendre (2017)*  **A systematic review of silver diamine fluoride: Effectiveness and application in older adults.** | 3 studies  895 (266-323) | Hong Kong 3 | 2010-2016 | Only 3 papers were included (all 3 studies were high quality and had a low degree of bias).  There were no studies investigating the effect of SDF on coronal caries in adults. | Black staining was reported but no serious adverse events were observed. | This systematic review evaluates the use of SDF for both root caries prevention and arrest in older adults. Existing reports of SDF trials support effectiveness in root caries prevention and arrest, remineralization of deep occlusal lesions and treatment of hypersensitive dentin. | **√** |  |  |  |  |  | There was no referral to a priori designed protocol.  The authors did not declare that there was no conflict of interests.  Only English papers were included.  There were some differences in the placebos and the use of Oral Hygiene Instruction and Education, application frequencies. | Dentin hypersensitivity is brought into the conclusions but has not been included in the search. The authors mentioned in the discussion that they expanded their search to include SDF safety, remineralization and desensitization studies in adults but there are no details given on this in the paper. The authors reported the differences of effect sizes independently for each study and did not attempt to combine them into an overall effect size. This was likely to be appropriate because of the study duration, differences in placebos. |  |
| *Oliveira (2018a)*  **Controlling caries in exposed root surfaces with silver diamine fluoride: A systematic review with meta-analysis** | 4 articles - 3 trials  895 (266-323) | Hong Kong 3 | 2010-2017 | The low number of clinical trials.  All included trials were from the same group of investigators and enrolled Chinese older adults with a low risk of developing caries.  Moderate to considerable statistical heterogeneity. | Black staining was reported but the complaints about it were rare among older adults. | Annual 38% SDF applications are effective against caries initiation and progression. The preventive effect is similar to that of 5% fluoride varnish and 1% chlorohexidine varnish. Further research is needed to replicate these findings and to determine the best frequency and intervals of SDF applications. | **√** |  |  |  |  |  | All covered by the authors. | Well conducted systematic review; the search was comprehensive including searching for on-going trials, theses and dissertations. The authors attempted to contact studies’ authors to obtain missing information. It referred to a priori designed protocol. However, its status in PROSPERO had not been updated and still “ongoing” although it has been completed and published. |  |

* Quality assessment: AR: Assessing the relevance between the overview and the review being assessed, SEC: Study Eligibility Criteria, ISS: Identification and Selection of Studies, DCSA: Data Collection and Study Appraisal, SF: Synthesis and Findings, RoB: Risk of Bias;

Low risk: high risk; unclear risk;

** Some reviews did not state the countries of origin of the included studies, but this was determined by comparing with the overlapping studies across reviews or by going back to the individual studies and written in an underlined text.

| **Additional file 3B. Coronal caries reviews’ findings and quality appraisal** | | | | | | | | | | | | | | | | |
| --- | --- | --- | --- | --- | --- | --- | --- | --- | --- | --- | --- | --- | --- | --- | --- | --- |
| **Systematic review** | **Number of studies**  **Sample size (range)** | **Type of SDF studies** | | **Countries in which studies were conducted** | **Publication date range** | **Review authors’ stated limitations** | **Adverse events** | **Review authors' stated conclusions** | **Quality assessment*** | | | | | | **Overview authors’ additional limitations** | **Overview authors’ comments** |
|  |  |  |  |  |  |  |  |  | **AR** | **Review Process** | | | | **RoB** |  |  |
|  |  | **RCT** | **Prospective CT** |  |  |  |  |  |  | **SEC** | **ISS** | **DCSA** | **SF** |  |  |  |
| *Rosenblatt (2009)*  **Silver diamine fluoride: a caries “silver-fluoride bullet”** | 2  827 (375-452) | 2*** |  | Not stated**  *Cuba 1*  *Hong Kong 1* | 2002-2005 | “Neither study provided a power calculation. This, therefore, is a limited dataset upon which to build a new preventive strategy.” “Only one of the two identified and qualifying study extended their research to permanent teeth. One study only examined only maxillary interior, and not posterior teeth. This limits the data upon which one might base clinical application of SDF.” | There was no significant difference between the control and experimental  groups in pulpal incident. Staining was similar in both control and experimental groups, and troubled 7% of participants in one trial.  SDF caused 24-hour tissue sensitivity in three of the 153 participants in one trial. The hypothetical risks of possible toxicity to the pulp were not supported. | “SDF can have a significant and substantial benefit in arresting and preventing caries. By implication, SDF could provide a new quantitative preventive benefit for individuals and populations.” | **√** |  |  |  |  |  | Small number of included studies.  The authors did not declare that there was no conflict of interests.  There was no referral to a priori designed protocol. | There is limited evidence to support the effectiveness of SDF in preventing/arresting caries because the small number of included studies. Moreover, one study should not have been included because it compared the effectiveness of SDF to water, while the research question is clearly stated:" Will SDF more effectively prevent caries than fluoride varnish?" |
| *Duangthip (2015)*  **Non-surgical treatment of dentin caries in preschool children - systematic review** | 4 - 3 SDF studies  678 (91-375) | 3 |  | China 2  Brazil 1 | 2002-2012 | “Few studies were included in the review.”  “Only English papers were reviewed, and this may lead to a reporting bias.”  “Some included studies were assessed as at moderate or high risk of bias”  “Although the inter- and intra-reliability of all included studies in this review were high, a major concern is a lack of an accepted clinical gold standard which can reliably differentiate between active and arrested lesions.”  “It should be noted that papers of co-authors of this review were finally included and there may be bias.” | Black staining was reported. However, it did not lead to an increase in parental dissatisfaction.  Major adverse effects were neither systematically studied nor reported in the included studies. | “There is limited evidence to support the effectiveness of SDF applications once/twice a year and that of daily tooth brushing with fluoride toothpaste in arresting or slowing down the progression of active dentin caries in primary teeth in preschool children. More well-designed RCTs are required to confirm these findings.” | **√** |  |  |  |  |  | There was no referral to a priori designed protocol. | Only 3 English studies were included (2 at low risk of bias and 1 at a high risk of bias), which limits the evidence regarding the effectiveness of SDF in arresting dentine caries in primary teeth. |
| *Gao (2016a)*  **Clinical trials of silver diamine fluoride in arresting caries among children: a systematic review** | 19  some trials did not report the sample size | 7 | 12**** | Brazil 5  China 5  Japan 4  Hong Kong 2  Nepal 1  Cuba 1  Argentina 1 | 1969-2016 | “It is noteworthy that the reliability of some studies included in this review was relatively low because most of the clinical studies on SDF were conducted before the Consolidated Standards of Reporting Trials (CONSORT) statement was developed.” | Black staining was reported but no serious adverse events were reported. | “SDF was commonly used at a high concentration (38%, 44,800 ppm fluoride) and is effective in arresting dentine caries in primary teeth. There is no consensus on its number and frequency of application to arrest caries. Further studies are necessary to develop evidence-based guidelines on its use in children.” | **√** |  |  |  |  |  | Studies conducted before 2002 were at low quality and there was no referral to a priori designed protocol. | The authors have included the countries' languages where most of SDF studies are likely to have taken place and the search was quite comprehensive in terms of coverage of databases. In conclusion, this SR included 19 studies of different languages and showed that 38% SDF is effective in arresting dentin caries in primary teeth. |
| *Gao (2016b)*  **Caries remineralisation and arresting effect in children by professionally applied fluoride treatment – a systematic review** | 17 - 7 SDF trials  2548 (60-976) | 7 |  | Not stated**  *China 2*  *Brazil 2*  *Hong Kong 1*  *Cuba 1*  *Nepal 1* | 2001-2012 | Limited number of RCTs. The methodology and outcome measurement varied between studies. Blinding of outcome measurement and allocation concealment were either not achieved or not mentioned by some researchers. The sample size of some studies was small, while some studies did not report the statistical procedure of sample size calculation or justified the sample size used in their studies. Only English articles were included which makes the review probably not comprehensive. | Black staining was reported, which may cause dissatisfaction of the children and their parents.  No serious adverse events were reported | 38% SDF is effective in arresting active dentine caries.” | **√** |  |  |  |  |  | There was no referral to a priori designed protocol. | Considering the several limitations this review has, SDF has been found to be effective in arresting dentine caries based on 6 RCTs.  Only one study assessed the effectiveness of SDF in remineralising early enamel caries. Therefore, it was not sufficient to build an evidence. |
| *Contreras (2017)*  **Effectiveness of silver diamine fluoride in caries prevention and arrest: a systematic literature review** | 7  3043 (22-1016) | 7 |  | Brazil 2  Cuba 1  China 1  Philippines 1  Hong Kong 1  Nepal 1 | 2005-2016 | The review analyzed only manuscripts written in English, which could have introduced bias into the analysis.  An analysis of bias was not conducted.  Trial reporting of the assessed studies could suggest additional biases such as selection, detection, and other biases. Differences between examiner criteria or stringency of the examiner limited a comparative analysis of caries arrest detection.  Inclusion and exclusion criteria were not defined in all studies.  The determination of the arrested caries was not consistent across studies.  The randomization and allocation process were not clearly stated in most of studies. | Black staining and the development of reversible, small white lesions in the oral mucosa were reported. | SDF, at concentrations of 30% or 38% is more effective than other preventive management strategies for arresting dentinal caries in the primary dentition and shows potential as a caries preventive treatment in primary teeth and permanent first molars. Standardized SDF protocols must be developed to allow meaningful study comparisons and establish treatment guidelines. | **√** |  |  |  |  |  | There was no referral to a priori designed protocol. | The authors did not declare that there was no conflict of interests.  This systematic review has some limitations, but it shows that SDF 30% and 38% is effective in preventing and arresting caries in the primary dentition and permanent first molars. |
| *Chibinski (2017)*  **Silver Diamine Fluoride Has Efficacy in Controlling Caries Progression in Primary Teeth: A Systematic Review and Meta-Analysis** | 11  4089 (60-1016) | 11 |  | Not stated**  *Brazil 3*  *China 2*  *Hong Kong 2*  *Cuba 1*  *Nepal 1*  *Philippines 1*  *Turkey 1* | 2002-2016 | The most remarkable feature of the included papers is the great variation of the measured outcomes. The outcomes were: (1) number of inactive carious surface (2) number of active carious surface (3) dmft index (4) number of teeth with inactive carious lesions (5) number of teeth with new carious lesions  ;(6) number of inactive carious lesions in the first permanent molars. | Not reported | SDF is more effective than other active treatments or placebo for caries arrestment in primary teeth. The body of evidence was of high quality for primary teeth. There is not enough evidence to draw a conclusion about caries arrestment in first permanent molars. | **√** |  |  |  |  |  | All covered by the authors | Well conducted systematic review; the authors searched the grey literature, dissertations and thesis, and contacted the authors of some studies for additional details, and it referred to *a priori* designed protocol. However, its status in PROSPERO had not been updated and still “ongoing” although it has been completed and published.  This supports the hypothesis that SDF is effective in arresting caries in primary teeth. |
| *Oliveira (2018b)*  **The Effect of Silver Diamine Fluoride in Preventing Caries in the Primary Dentition: A Systematic Review and Meta-Analysis** | 4  1038 (91-452) | 4 |  | Brazil 2  China 1  Cuba 1 | 1991-2012 | Although authors performed a comprehensive search, a very small number of clinical trials having the development of new caries lesions as outcome mea­sure, was found. The trials identified for this review were primarily designed to investigate SDF for dental caries arrest and most applied SDF to carious lesions only. | The development of small, mildly painful white lesions in the mucosa due to inadvertent contact with SDF solution that healed spontaneously within 48 hours.  The pa­rental satisfaction with children’s dental appearance at 24-month follow up was similar between experimental and control groups.  There was no significant difference between the control and experimental  groups in pulpal incident. Postulated adverse events such as discomfort associated with SDF use; metallic taste, burning sensation, acute or chronic toxicity, or allergic reactions were not reported. | SDF topical applications may have a significant impact on reducing the burden of untreated caries in children. Nevertheless, the evidence on the preventive effect of SDF is based on only 2 small positive clinical trials with important limitations regarding study design and implementation. More rigorously designed studies, reported according to the CONSORT statement, are warranted to ensure unbiased high-qual­ity evidence on the benefits of SDF applications for caries prevention. | **√** |  |  |  |  |  | All covered by the authors | Well conducted systematic review; the search was comprehensive including searching for on-going trials, theses and dissertations. The authors attempted to contact studies authors to obtain missing information.  It referred to *a priori* designed protocol. However, after going back to the registered protocol in PROSPERO, a deviation was noted between the protocol and the actual review, but it was justified after contacting the authors of the systematic review. Moreover, its status in PROSPERO had not been updated and still “ongoing” although it has been completed and published. |

* Quality assessment: AR: Assessing the relevance between the overview and the review being assessed, SEC: Study eligibility criteria, ISS: Identification and selection of studies, DCSA: Data collection and study appraisal, SF: Synthesis and findings, RoB: Risk of bias;

Low risk: high risk; unclear risk;

** Some reviews did not state the countries of origin of the included studies, but this was determined by comparing with the overlapping studies across reviews or by going back to the individual studies and written in a underlined text.

*** Chu study was defined as cohort design in this review while it was included as RCT in the other reviews. However, by going back to the study, it turned out that it is not a cohort design, but not clearly defined as an RCT as well.

**** The review stated that they included 19 prospective clinical trials without further clarification about which of them were RCTs. However, 7 of the reviews were determined as RCTs by the overlapping in studies across reviews.
