# Supplementary material for: Silver diamine fluoride for managing carious lesions: an umbrella review
Source: BMC Oral Health. 2019 Jul 12;19:145. doi: 10.1186/s12903-019-0830-5 (PMC6626340; doi:10.1186/s12903-019-0830-5)
Supplement: Supplementary file 4 — Characteristics of reviews related to outcomes, outcome measures and results. (DOCX 40 kb) [file 12903_2019_830_MOESM4_ESM.docx]

| Additional file 4. Characteristics of reviews related to outcomes, outcome measures and results | | | | | | | | | | | | | | | | | | |
| --- | --- | --- | --- | --- | --- | --- | --- | --- | --- | --- | --- | --- | --- | --- | --- | --- | --- | --- |
| Outcome | **Review** | **Intervention** | | | **Comparator** | | | **Duration/follow up** | | | **Outcome measures with results** | | | | | | **No. of studies used in synthesising these results**** | **Summary***** |
|  |  | **Concentration** | | **Frequency of application** | **FV** | **ART restorations/GIC** | **Placebo/no treatment*** | **One year** | **Two years** | **Three years** | **Success rates** | **Prevented fraction (PF)** | **Mean difference (MD) in the changes of DMFRS/DFRS** | **Risk ratio**  **(RR)** | **Weighted mean difference (WMD)** | **Numbers needed to treat (NNT)** |  |  |
|  |  | 30%  SDF | 38%  SDF |  |  |  |  |  |  |  |  |  |  |  |  |  |  |  |
| Root caries prevention | **Gluzman, 2013** | × | ✓ | Annual | × | × | ✓ | × | × | ✓ | 72% compared to placebo | ______ | ______ | ______ | ______ | ______ | 1 study | **Success rate**  One review reported success rate for annually-applied SDF at 36 months 72%.  **MD**  One review measured MD for SDF compared to placebo at (24-36) months intervals, MD= -0.33.  **PF**  Two reviews compared SDF to placebo at (12-36) months intervals, PF=25-71%. |
|  | **Wierichs, 2015** | × | ✓ | Different frequencies | × | × | ✓ | Different follow up durations | | | ______ | ______ | MD= -0.33 (95% CI= -0.39, -0.28) at 24-36 months | ______ | ______ | ______ | 2 studies |  |
|  | **Hendre, 2017** | × | ✓ | Different frequencies | × | × | ✓ | Different follow up durations | | | ______ | PF= 25% in a 24-month study  PF= 71% in a 36-month study | ______ | ______ | ______ | NNT= 3.3 in a 24-month study  NNT= 2.5 in a 36-month study | 1 study at 24-month follow-up  1 study at 36-month follow-up |  |
|  | **Oliveira, 2018a** | × | ✓ | Different frequencies | × | × | ✓ | Different follow up durations | | | ______ | PF= 68% (95% CI= 54-83%) at 12 months  PF= 50% (95% CI= 37-63%) at 24 months  PF= 60% (95% CI= 45-57%) at ≥ 30 months | ______ | ______ | WMD= -0.48 (95% CI= -0.69 to -0.27) at 12 months  WMD= -0.56 (95% CI= -0.77 to -0.36) at 24 months  WMD= -0.80 (95% CI= -1.19, to 0.42) at ≥ 30 months | ______ | 2 studies at 12-month follow-up  3 studies at 24-month follow-up  2 studies at ≥ 30-month follow-up |  |
| Root caries arrest | **Hendre, 2017** | × | ✓ | Different frequencies | × | × | ✓ | Different follow up durations | | | ______ | PF was 725% greater than placebo at 24 months  PF was 100% greater than placebo at 30 months | ______ | ______ | ______ | NNT= 1.8 in a 30-month study | 1 study at 24-month follow-up  1 study at 30-month follow-up | **PF**  One review reported that PF for SDF was (100%-725%) greater than placebo at (24-30) months intervals. |
| Coronal caries prevention**** | **Rosenblatt, 2009** | × | ✓ | Different frequencies | ✓ | × | ✓ | Different follow up durations | | | ______ | Lowest PF= 70.3% | ______ | ______ | ______ | Highest NNT 0.9 (95% CI = 0.4- 1.1) | 2 studies | **PF**  Two reviews compared SDF to placebo at different time intervals, PF=70-78%.  GIC was reported to be more effective than SDF at 12 months in one review, PF= -6%, but the difference was not statistically significant. |
|  | **Oliveira, 2018b** | Different concentrations | | Different frequencies | ✓ | ✓ | ✓ | Different follow up durations | | | ______ | Placebo: PF = 77.5% (95% CI= 68-78%) at ≥ 24 months  FV: PF = 54.0% (95% CI= 27-73%) at ≥ 24 months  GIC: PF= –6.09% (95% CI= -36-16%) at 12 months | ______ | ______ | Placebo: WMD = –1.15 (95% CI= -1.48 to -0.82) at ≥ 24 months  FV: WMD = –0.43 (95% CI= -0.70 to -0.16) at ≥ 24 months  GIC: WMD= 0.34 (95% CI= -1.02 to 1.70) at 12 months | ______ | 2 studies comparing SDF to placebo  1 study comparing SDF to FV  1 study comparing SDF to GIC |  |
| Coronal caries arrest**** | **Rosenblatt, 2009** | × | ✓ | Different frequencies | ✓ | × | ✓ | Different follow up durations | | | ______ | lowest PF 96.1% | ______ | ______ | ______ | Highest NNT 0.8 (95% CI = 0.5-1.0) | 2 studies | **Success rates**  Four reviews reported success rates. These ranged from SDF: (65-91%); FV: (38-44%); GIC: (39-82%); placebo: 34% at (6-36) months intervals.  **PF**  One review reported that the lowest PF for SDF compared to FV and placebo in the included studies was 96.1%.  **RR**  One review included 2 studies that compared SDF  to FV or ART restorations. (RR=1.66).  It included 2 studies that compared SDF to placebo/no treatment (RR=2.54). |
|  | **Duangthip, 2015** | Different concentrations | | Different frequencies | ✓ | ✓ | ✓ | Different follow up durations | | | SDF (65-91%) compared with no treatment (34%), FV (38-44%) and interim GIC (39-82%). | ______ | ______ | ______ | ______ | ______ | 3 Studies |  |
|  | **Gao, 2016a** | × | ✓ | Different frequencies | ✓ | ✓ | ✓ | Different follow up durations | | | 86% (95% CI= 47-89%) at 6 months  81% (95% CI= 59-93%) at 12 months  78% (95% CI= 70-85%) at 18 months  65% (95% CI= 35-86%) at 24 months  71% (95% CI= 65-83%) at ≥ 30 months | ______ | ______ | ______ | ______ | ______ | 5 studies at 6-month follow-up  6 studies at 12-month follow-up  4 studies at 18-month follow-up  4 studies at 24-month follow-up  4 studies at ≥ 30-month follow-up |  |
|  | **Gao, 2016b** | × | ✓ | Different frequencies | ✓ | ✓ | ✓ | Different follow up durations | | | 66% (95% CI= 41-91%) | ______ | ______ | ______ | ______ | ______ | 5 Studies |  |
|  | **Chibinski, 2017** | Different concentrations | | Different frequencies | ✓ | ✓ | ✓ | Different follow up durations | | | ______ | ______ | ______ | Active treatment: RR= 1.66 (95% CI= 1.41–1.96)  Placebo: RR= 2.54 (95% CI=1.67–3.85). | ______ | ______ | 2 studies comparing SDF to active materials  2 studies comparing SDF to placebo |  |

* No treatment and placebo were combined.

** This might differ from the number of included studies in some reviews because synthesising all included studies’ results was not always possible.
*** The summary presents various outcomes where meaningful results could be combined.

**** Contreras et al, systematic review was not included because they only reported the original studies individually.
